# Factors Associated with Pain During Transperineal Prostate Fusion Biopsy Under Local Anaesthesia: A Prospective Study

**DOI:** 10.3390/diagnostics16193195

**Published:** 2026-10-01

**Authors:** Bartłomiej Marczak, Katarzyna Skrobisz, Aleksandra Gostomska, Justyna Klecha, Kevin Miszewski, Maciej Dolny, Dorota Szczodrowska, Anna Koelmer, Amir Naziri, Adam Kaftan, Marcin Matuszewski

**Affiliations:** 1 Department of Urology, University Clinical Centre, Medical University of Gdańsk, Smoluchowskiego 17, 80-214 Gdańsk, Poland; 2 Department of Radiology, University Clinical Centre, Medical University of Gdańsk, Smoluchowskiego 17, 80-214 Gdańsk, Poland; 3 Faculty of Medicine, Medical University of Gdańsk, Marii Skłodowskiej-Curie 3a, 80-210 Gdańsk, Poland; 4 Centre of Biostatistics and Bioinformatics Analysis, Medical University of Gdańsk, Dębinki 1, 80-211 Gdańsk, Poland

**Keywords:** prostate biopsy, prostate fusion biopsy, transperineal biopsy, procedural pain, anxiety, local anaesthesia, magnetic resonance imaging, patient-reported outcomes

## Abstract

**Background**/**Objectives**: Transperineal MRI-fusion prostate biopsy under local anaesthesia is increasingly performed in the outpatient setting, which places greater weight on procedural tolerability. Psychological and anatomical determinants of pain have rarely been compared within a single model, and MRI-derived perineal anatomical parameters have not been evaluated. We aimed to identify factors associated with procedural pain and to determine at which step pain peaks. **Methods**: In this prospective, pre-registered, single-centre cohort study (OSF: osf.io/pbacu), 182 consecutive men referred for transperineal MRI–ultrasound fusion biopsy under local anaesthesia were assessed for eligibility, and 177 constituted the Full Analysis Set. State anxiety was measured with the STAI-S before and after biopsy. Pain was rated on a Numeric Rating Scale (NRS 0–10) by a nurse not involved in the procedure at four predefined steps: probe insertion, skin infiltration, periprostatic nerve block and core sampling. Midline perineal tissue thickness (MPT) and levator ani thickness were measured on pre-biopsy T2-weighted MRI by a radiologist blinded to outcomes. The primary outcome was the maximum NRS score; ten candidate predictors were entered simultaneously into a multivariable linear regression model. **Results**: Clinically significant pain (maximum NRS score ≥ 4) was reported by 157 of 177 men (88.7%). Pain differed markedly across steps (F(2.68, 472.39) = 230.51, *p* < 0.001, partial η^2^ = 0.567) and peaked at the periprostatic nerve block (5.88 ± 2.03), exceeding core sampling by 2.62 points (95% CI 1.85–3.40). In multivariable analysis (R^2^ = 0.192), pre-biopsy anxiety was the strongest independent predictor (standardised β = 0.30, partial R^2^ = 0.094, *p* < 0.001), followed by prostate volume (β = 0.19, *p* = 0.014); MPT showed only an exploratory, borderline association (β = 0.17, *p* = 0.052). Core number, body mass index and levator ani thickness were not associated with pain. **Conclusions**: Most men undergoing transperineal MRI-fusion biopsy experience clinically significant pain, peaking at administration of the periprostatic block rather than at core sampling. Pre-procedural anxiety and prostate volume, both available beforehand, support simple risk stratification, while the block itself is the most logical target for analgesic improvement.

## 1. Introduction

Prostate cancer remains one of the most prevalent malignancies in men. Lesions deemed suspicious on MRI should undergo targeted sampling [1,2,3].

Transperineal biopsy is associated with significantly lower rates of infection and sepsis while providing comparable detection of clinically significant prostate cancer [4,5,6]. The feasibility of transperineal biopsy under local anaesthesia has facilitated its implementation in the outpatient setting, improving accessibility while placing greater emphasis on patient-reported tolerability [7]. Transperineal biopsy is nevertheless often associated with higher patient-reported pain than the transrectal approach, particularly during anaesthetic infiltration [8,9].

Psychological factors are among the best-established determinants of procedural pain. Anxiety and stress have been classified as probable predictors of acute postsurgical pain [10], and postoperative pain intensity [11]. In prostate biopsy, however, anxiety is rarely compared with anatomical determinants within a single model.

Anatomical predictors have received less attention, and the available evidence derives largely from the transrectal route, where prostate–anus surface distance, anorectal angle and total prostate volume correlate with procedural pain [12].

In the transperineal setting, predictors reported to date have been clinical or procedural rather than anatomical: severe anxiety, younger age and longer procedural duration [13], and the number of biopsy cores [14]. To our knowledge, MRI-derived perineal anatomical parameters have not previously been evaluated in relation to procedural pain.

Perineal soft-tissue thickness, as an anatomical surrogate of the tissue traversed by the biopsy needle, may therefore represent an additional determinant of procedural pain. Identifying robust predictors could support risk-stratified analgesia, allowing enhanced anaesthetic measures to be directed towards the men most likely to require them.

The aim of this prospective study was to evaluate psychological, clinical and MRI-derived anatomical factors associated with pain during transperineal prostate biopsy performed under local anaesthesia.

## 2. Methods

### 2.1. Study Design, Registration, and Setting

This was a prospective, single-centre observational study conducted at the Department of Urology of a single tertiary care centre. A total of 181 consecutive patients were prospectively enrolled between May 2025 and July 2026. The study protocol was approved by the Bioethics Committee for Scientific Research of the Medical University of Gdańsk (approval number: KB/254/2025; 9 May 2025), and all participants provided written informed consent prior to inclusion, including consent for publication of anonymised data. This study was conducted in accordance with the Declaration of Helsinki and was reported in accordance with the STROBE guidelines for observational studies.

This study was pre-registered on the Open Science Framework (OSF) prior to multivariable analyses of the complete dataset (https://osf.io/pbacu, accessed on 27 September 2026). No multivariable analyses or anatomical measurements had been undertaken prior to registration. Refinements to the original protocol made during the conduct of the study are documented in Protocol Amendment on the OSF.

### 2.2. Study Population

Consecutive male patients referred for transperineal prostate biopsy were screened for eligibility.

Patients were eligible if they were male, aged 40 years or older, had a clinical indication for transperineal prostate biopsy (elevated PSA, abnormal digital rectal examination, and/or a PI-RADS ≥ 3 lesion on multiparametric MRI), were able to provide written informed consent, and had a pre-biopsy multiparametric MRI available for anatomical measurements.

Patients were excluded if they had contraindications to transperineal biopsy (such as active urinary tract infection or coagulopathy), were unable to complete the study questionnaires owing to cognitive impairment or a language barrier, or had undergone previous radical prostatectomy or pelvic radiotherapy.

### 2.3. Clinical and Demographic Variables

The following baseline variables were collected: age, body mass index (BMI), serum PSA, prostate volume, history of previous prostate biopsy and index lesion location. The index lesion was defined as the lesion with the highest PI-RADS score; in case of ties, the lesion with the largest maximum diameter was selected.

### 2.4. MRI Protocol and Anatomical Measurements

All patients underwent multiparametric MRI (mpMRI) of the prostate prior to biopsy as part of standard clinical care. MRI examinations were reviewed by a single experienced radiologist blinded to patient-reported outcomes. Target lesions were prospectively delineated by this radiologist according to PI-RADS version 2.1, and these delineations were used to guide targeted sampling during fusion-guided transperineal biopsy.

The following anatomical parameters were measured on T2-weighted images according to a standardised operating procedure developed prior to assessment:

Midline perineal tissue thickness (MPT): Linear distance from the perineal skin surface to the outer prostatic capsule, measured in the midline sagittal plane. MPT is a standardised midline anatomical surrogate of perineal soft-tissue thickness; it is not a reconstruction of the actual needle trajectories used for the periprostatic block or for individual biopsy cores, which are paramedian and target-dependent.

Levator ani thickness (LAT): The greatest perpendicular thickness of the levator ani muscle at the level of the expected paramedian needle path, measured separately on the left and right sides at the point of maximal thickness in the coronal plane, following radiology team consensus on optimal visualisation.

**Figure 1:**
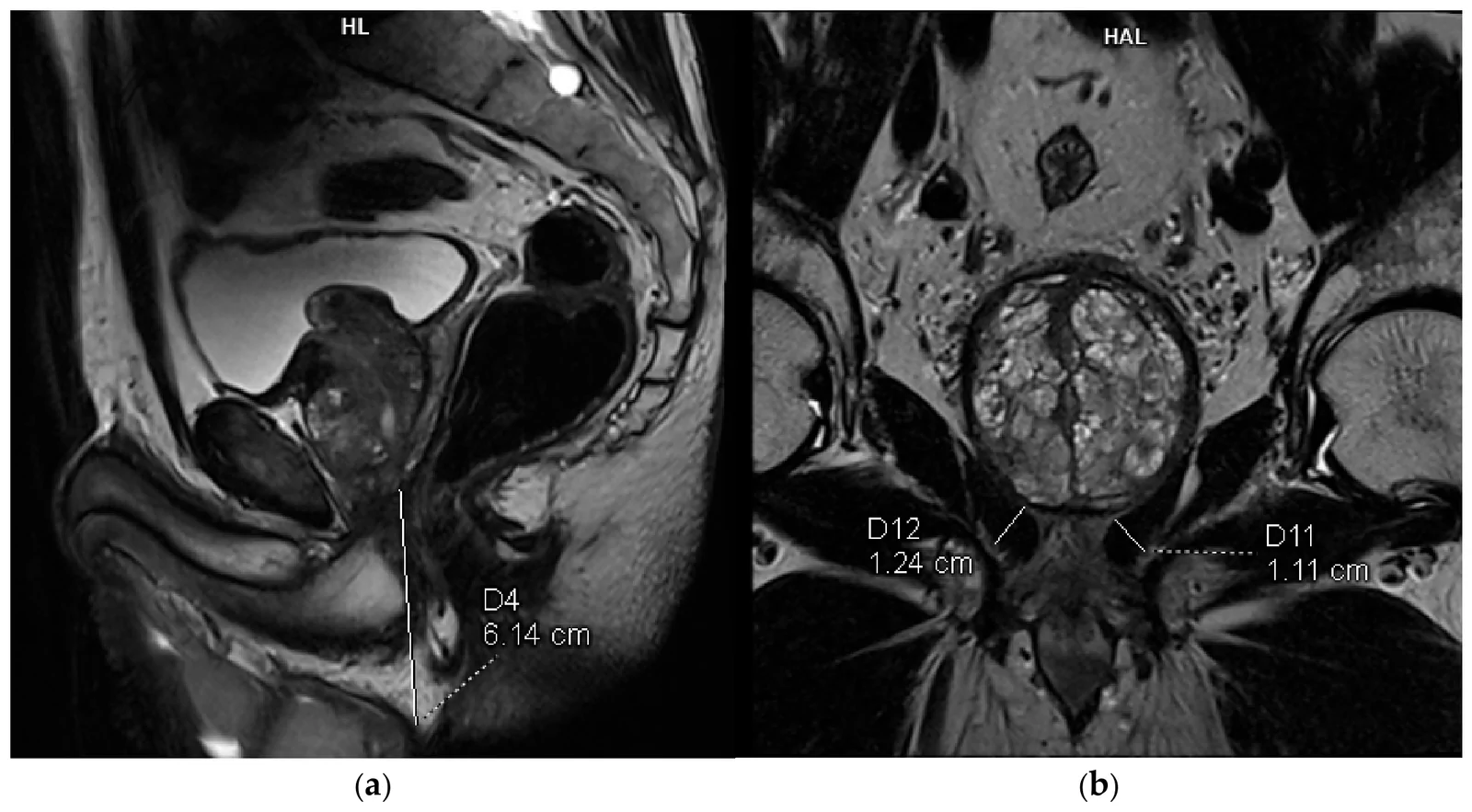
Standardised MRI measurements: (**a**) Midline sagittal plane: midline perineal tissue thickness (MPT), the linear distance from the perineal skin surface to the outer prostatic capsule. (**b**) Coronal plane: levator ani thickness (LAT), the greatest perpendicular thickness of the levator ani muscle at the level of the expected paramedian needle path, measured separately on the right and left sides.

Both measurements are illustrated in Figure 1.

Inter-rater reliability was assessed by an independent second radiologist on a random subset of 18 cases.

### 2.5. Biopsy Procedure

All biopsies were performed transperineally under local anaesthesia in an outpatient setting by three urology residents (postgraduate years 3–6), each of whom had performed at least 20 transperineal biopsies before the start of the study, under the supervision of board-certified urologists. The operator was not recorded for individual procedures. Local anaesthesia was the only analgesic intervention; no oral analgesic, sedative or anxiolytic premedication was given. The operator was blinded to patients’ pre-procedural STAI-S scores at the time of the procedure.

**Figure 2:**
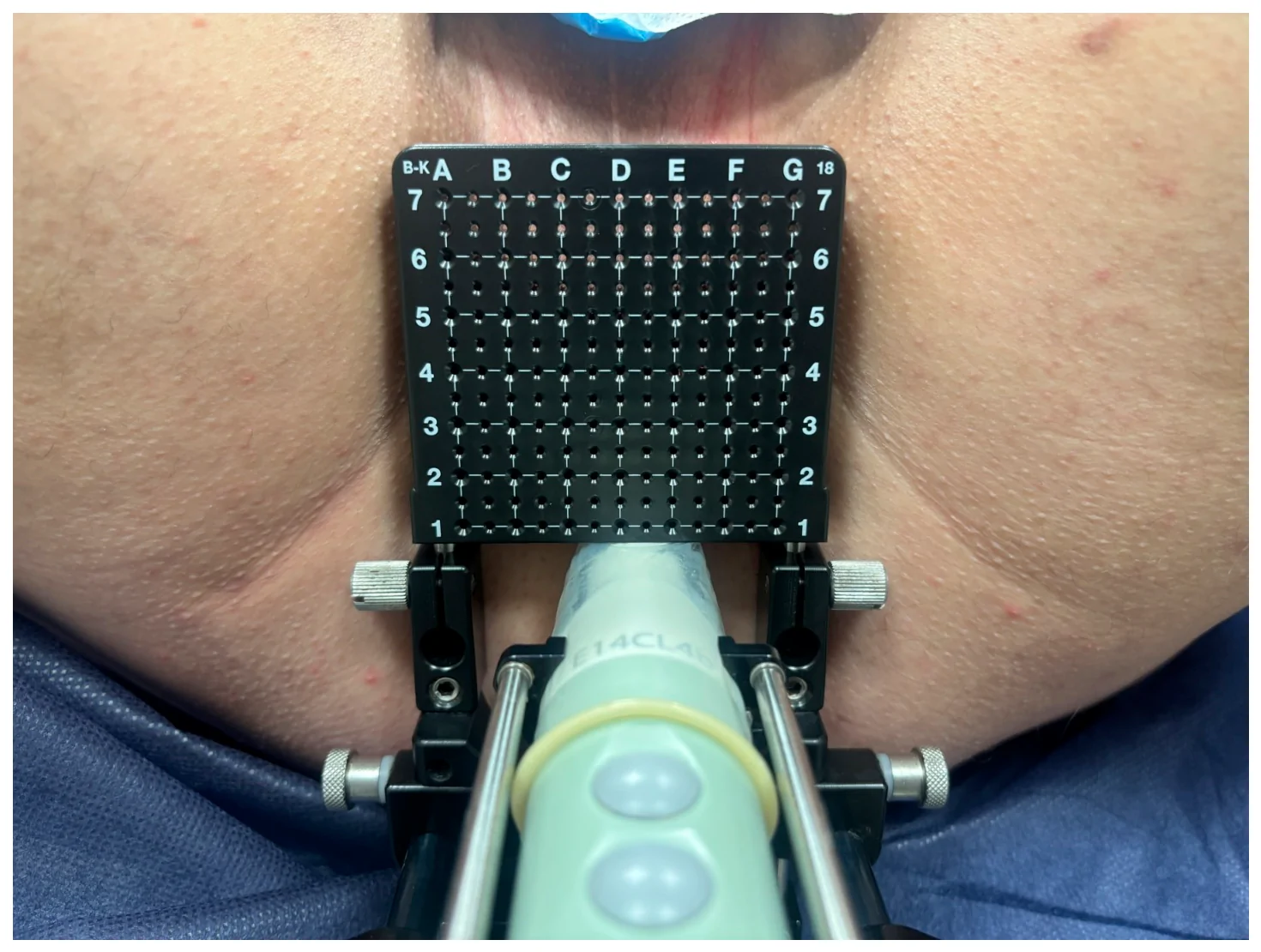
Procedural set-up: brachytherapy grid template mounted on a stepper. The letters and numbers on the template are the manufacturer’s grid coordinates.

MRI–ultrasound fusion-guided biopsies were performed using the BK 5000 ultrasound system (BK Medical ApS, Herlev, Denmark) equipped with a CIVCO stepper and brachytherapy template (CIVCO Medical Solutions, Coralville, IA, USA), using an 18 G × 25 cm Geotek Estacore biopsy needle (Geotek Medical, Ankara, Türkiye). The procedural set-up is shown in Figure 2.

Three to six cores were obtained per lesion, comprising targeted and perilesional cores from MRI-visible lesions; no systematic cores were taken. The total number of cores was therefore determined by the number and location of target lesions and was recorded for each patient.

Local anaesthesia consisted of perineal skin infiltration followed by a periprostatic nerve block, each with 10 mL of 1% lignocaine (20 mL in total). Skin infiltration was performed through two punctures, one on each side of the perineum, with approximately 5 mL infiltrated subcutaneously in a fan-like pattern on each side. The periprostatic block was administered under transrectal ultrasound guidance with a 22 G Chiba needle introduced through the same two entry sites (one puncture per side), parallel to the probe and visualised in the sagittal plane. On each side, 5 mL was deposited in small aliquots in three phases: along the needle track while advancing towards the apex; at the prostatic apex, lateral to the urethra; and in the periprostatic space along the lateral border of the gland, within the neurovascular bundle. An interval of 5 min was allowed between completion of the block and the first biopsy core.

### 2.6. Assessment of Anxiety

Pre-procedural anxiety was assessed using the State subscale of the State-Trait Anxiety Inventory (STAI-S; Polish adaptation by Wrześniewski, Sosnowski, Jaworowska, and Fecenec; published by Pracownia Testów Psychologicznych (PTP), Warsaw).

STAI-S was self-administered by the patient under standardised conditions in a quiet room, 10 min before entering the procedure room. It was repeated 5 min after completion of the biopsy.

Administration and scoring was done under the supervision of a co-investigator psychologist responsible for the psychometric component of this study.

### 2.7. Pain Assessment

Pain intensity was assessed using the Numeric Rating Scale (NRS; 0 = no pain and 10 = worst imaginable pain) by a nurse blinded to STAI-S scores. Pain was recorded at four predefined procedural stages in real time, immediately following each stage: ultrasound probe insertion, perineal skin infiltration, periprostatic nerve block, and biopsy sampling. At each stage, the nurse asked the patient to rate only the pain experienced during the step just completed; the rating was obtained during a brief pause after completion of that step and before the next step began. Pain after completion of the procedure was not assessed.

Primary outcome: Maximum NRS score recorded during the procedure, defined as the highest NRS value across the four predefined stages. The maximum pain score was selected as the primary outcome because it reflects peak procedural discomfort and is the most clinically relevant value from the patient’s perspective.

Secondary outcomes: NRS scores at individual procedural stages; clinically significant pain, defined as maximum NRS score ≥4 at any stage; change in STAI-S score between pre- and post-procedural assessment; willingness to undergo the same procedure again if clinically indicated (binary).

### 2.8. Sample Size

The sample size was determined a priori. To detect a Spearman correlation of ρ = 0.25 with α = 0.05 (two-sided) and 80% power, 123 participants were required (G*Power 3.1 (Heinrich Heine University Düsseldorf, Düsseldorf, Germany), exact test, bivariate normal model). For multivariable linear regression with the pre-specified predictor set, the 10–15 events-per-variable rule required 70–105 participants. A logistic regression of clinically significant pain was also planned, for which 140–210 participants were required under an assumed event rate of approximately 50%. A target of N = 200 was set, with a pre-specified minimum of N = 180.

The observed proportion of patients with clinically significant pain (88.7%) exceeded the rate assumed at the design stage, leaving 20 patients in the reference group. Because this precluded a stable logistic model with the pre-specified predictor set, clinically significant pain is reported descriptively, and the multivariable analysis was conducted on the continuous outcome as planned.

### 2.9. Statistical Analysis

Continuous variables are presented as medians with interquartile ranges and as means with standard deviations and categorical variables as counts and percentages.

Pain intensity across the four procedural steps was compared using repeated-measures analysis of variance with procedural step as the within-subject factor. Sphericity was assessed with Mauchly’s test and degrees of freedom corrected using the Greenhouse–Geisser estimate where required; partial eta-squared is reported as the effect size. Post hoc pairwise comparisons were performed on estimated marginal means with Holm adjustment. Pre- and post-procedural STAI-S scores were compared using a paired *t*-test.

The outcome for the predictor analyses was the highest NRS score across the four procedural steps. Univariable linear regression was performed for each candidate predictor, with global *p*-values adjusted across predictors using the Benjamini–Hochberg procedure. All candidate predictors were then entered simultaneously into a multivariable linear regression model without stepwise selection: pre-biopsy state anxiety (STAI-S), midline perineal tissue thickness, levator ani thickness, prostate volume, body mass index, age, serum PSA, number of biopsy cores, number of prior biopsies, and anterior lesion location.

The multivariable model reports unstandardised coefficients with 95% confidence intervals, standardised coefficients, *p*-values, partial R^2^ and variance inflation factors. Predictor importance was judged on standardised coefficients, partial R^2^ and confidence interval width rather than on *p*-values alone. Predictors whose association remained statistically significant after adjustment are described as independently associated with pain, denoting a conditional association rather than a causal effect.

All tests were two-sided with α = 0.05. Analyses were performed by an institutional statistician in R version 4.3.3 (R Foundation for Statistical Computing, Vienna, Austria), using the stats, rstatix and car packages; figures were produced with the ggplot2 and ggstatsplot packages.

## 3. Results

### 3.1. Study Population

**Figure 3:**
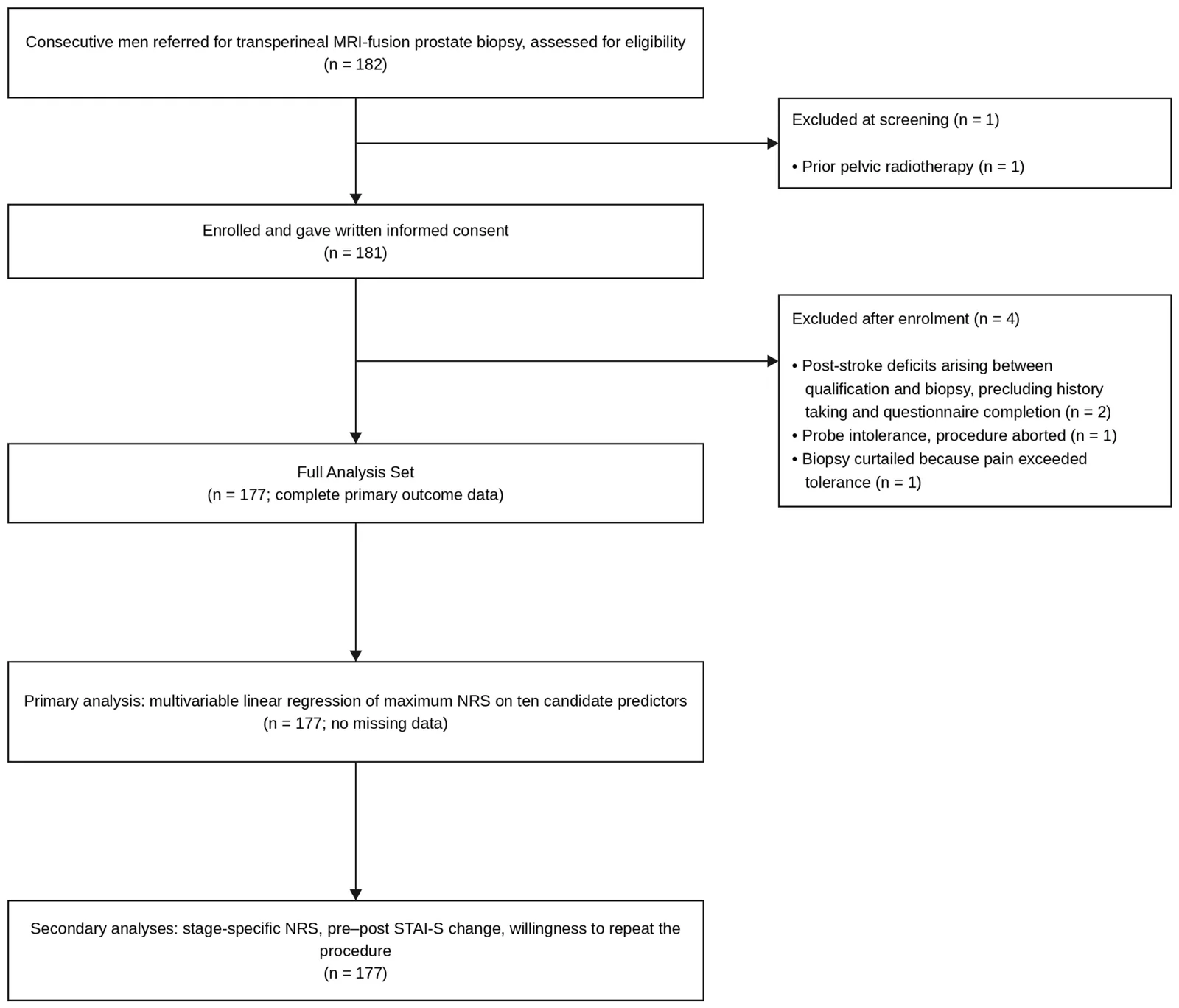
Participant flow through this study, from screening to the Full Analysis Set and the pre-specified analyses.

A total of 182 consecutive men referred for MRI–ultrasound fusion-guided transperineal prostate biopsy under local anaesthesia were assessed for eligibility. One was excluded at screening because of prior pelvic radiotherapy, a pre-specified exclusion criterion, and 181 were enrolled and gave written informed consent. Four were subsequently excluded: two developed post-stroke neurological deficits between qualification and the scheduled biopsy that precluded history taking and completion of the study questionnaires; in one the procedure was aborted because of intolerance to the ultrasound probe; and in one the biopsy was curtailed because intra-procedural pain exceeded the patient’s tolerance threshold. The Full Analysis Set comprised 177 patients with complete data for the primary outcome and all candidate predictors. Participant flow is shown in Figure 3. Baseline demographic, clinical, and procedural characteristics are summarised in Table 1.

### 3.2. Anxiety Before and After Biopsy

Mean pre-procedural STAI-S score was 43.3 ± 10.9 points (range 20–68); after the biopsy, the mean STAI-S score fell to 36.4 ± 10.6 points (range 20–79). The mean within-patient reduction was 6.9 points (95% CI 5.4–8.3; SD of differences 9.7), which was statistically significant on the paired *t*-test (t(176) = 9.42, *p* < 0.001). The corresponding effect size was moderate to large (Hedges’ g = 0.70, 95% CI 0.54–0.87), computed from the distribution of within-patient difference scores.

**Figure 4:**
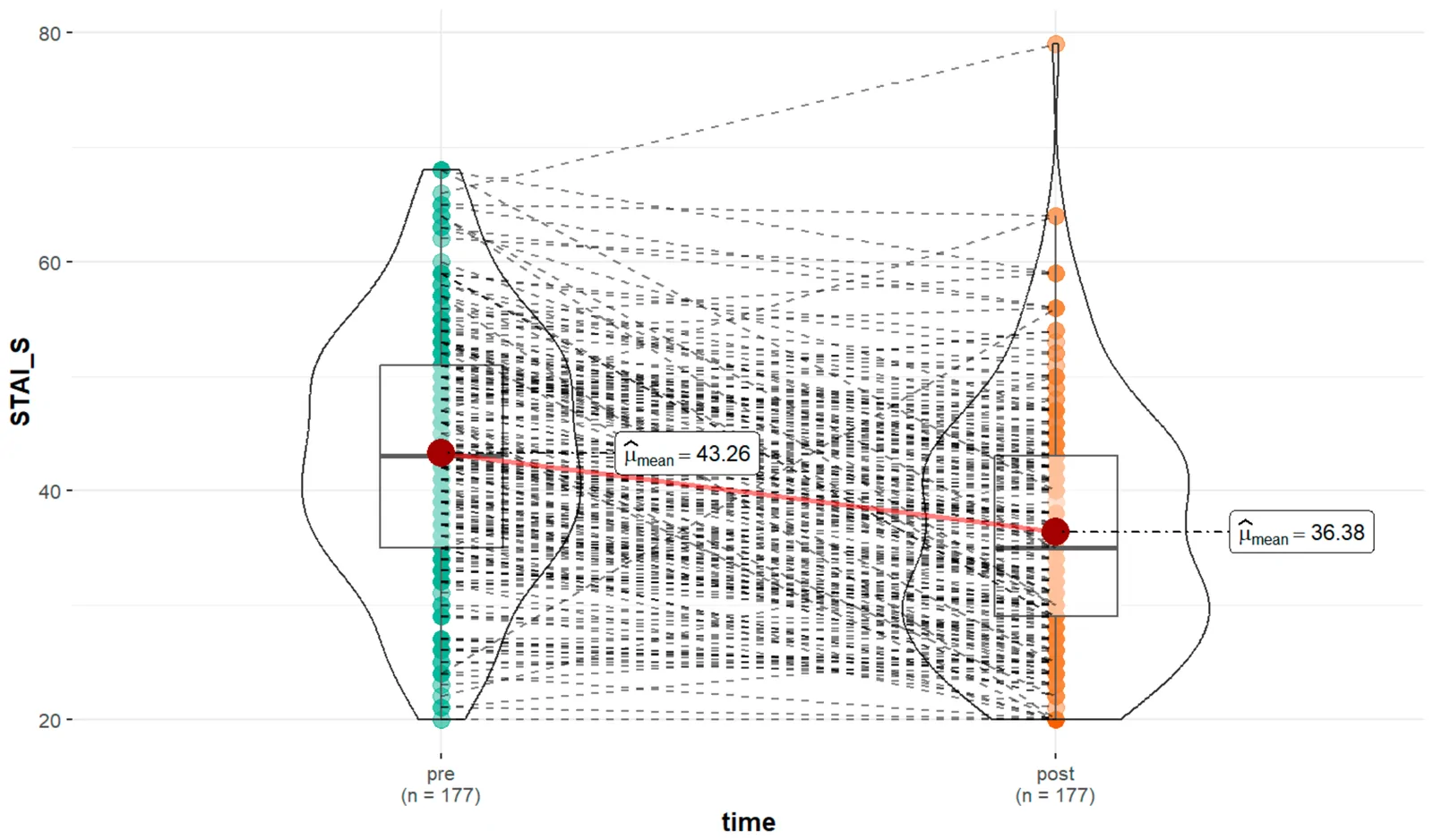
Pre- and post-procedural state anxiety (STAI-S) in the study cohort. Each coloured dot is the STAI-S score of one patient (teal, pre-procedural; orange, post-procedural); the colours denote the time point only and carry no further meaning. Dashed grey lines join the two assessments of the same patient. The outlines show the distribution of scores and the boxes the median with the interquartile range. Dark red points mark the group means, which are labelled and joined by the dash-dot line.

Anxiety decreased in 142 patients (80.2%), increased in 27 (15.3%) and was unchanged in 8 (4.5%). The post-procedural distribution retained a longer right tail than the pre-procedural one (maximum of 79 versus 68 points). Pre- and post-procedural scores were moderately correlated (r = 0.59), indicating that the ranking of patients by anxiety level was largely preserved across the two assessments and that the observed change reflected a downward shift in the whole distribution rather than regression among high scorers alone. Pre- and post-procedural STAI-S distributions are shown in Figure 4.

### 3.3. Pain Intensity During the Procedure

The primary outcome, the highest NRS score recorded across the four procedural steps, had a median of 6 (IQR 5–7) and a mean of 6.0 ± 2.0. Clinically significant pain (maximum NRS score ≥ 4) was reported by 157 of 177 patients (88.7%; 95% Wilson CI 83.2–92.6%) and severe pain (maximum NRS score ≥ 7) by 45.8%.

Pain was rated on a Numeric Rating Scale (NRS; 0–10) immediately after each of four predefined procedural steps: probe insertion, skin infiltration, periprostatic nerve block, and biopsy sampling.

**Figure 5:**
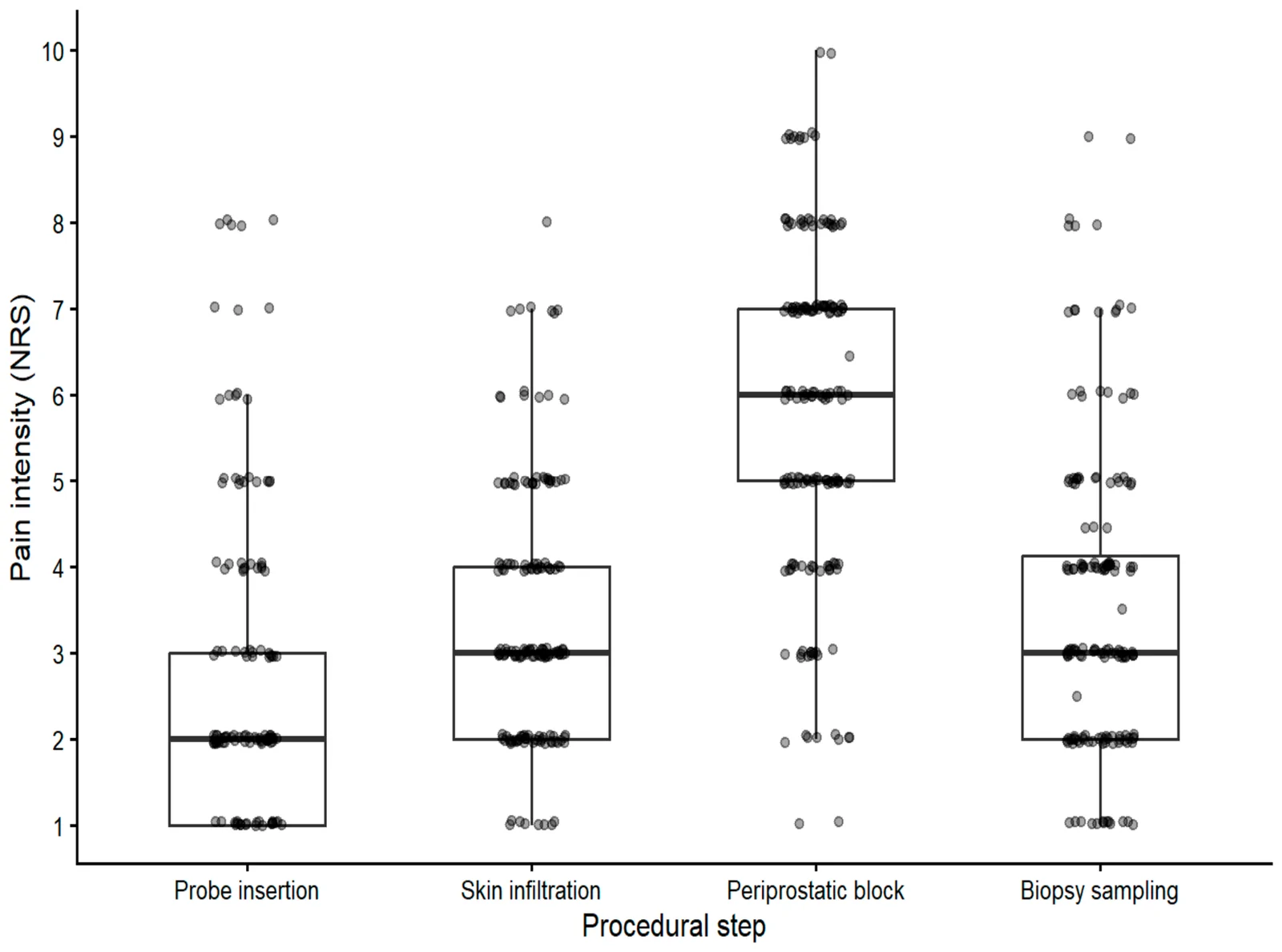
Pain intensity across the four procedural steps of transperineal MRI-fusion prostate biopsy under local anaesthesia (n = 177).

Mean pain across all steps was 3.66 ± 2.30. Pain scores at each step are summarised in Table 2 and Figure 5. Pain was the lowest at probe insertion (2.21 ± 1.91) and the highest at the periprostatic nerve block (5.88 ± 2.03); skin infiltration (3.22 ± 1.57) and biopsy sampling (3.31 ± 1.93) fell in between. Pain of at least moderate intensity (NRS score ≥ 4) was reported by 87.0% of patients during the block, compared with 20.9%, 36.2% and 41.2% during probe insertion, skin infiltration and biopsy sampling; severe pain (NRS score ≥ 7) occurred in 42.9% during the block.

Repeated-measures ANOVA showed a significant main effect of procedural step. Sphericity was violated (Mauchly’s test *p* < 0.001) and the Greenhouse–Geisser correction applied: F(2.68, 472.39) = 230.51, *p* < 0.001, partial η^2^ = 0.567.

**Figure 6:**
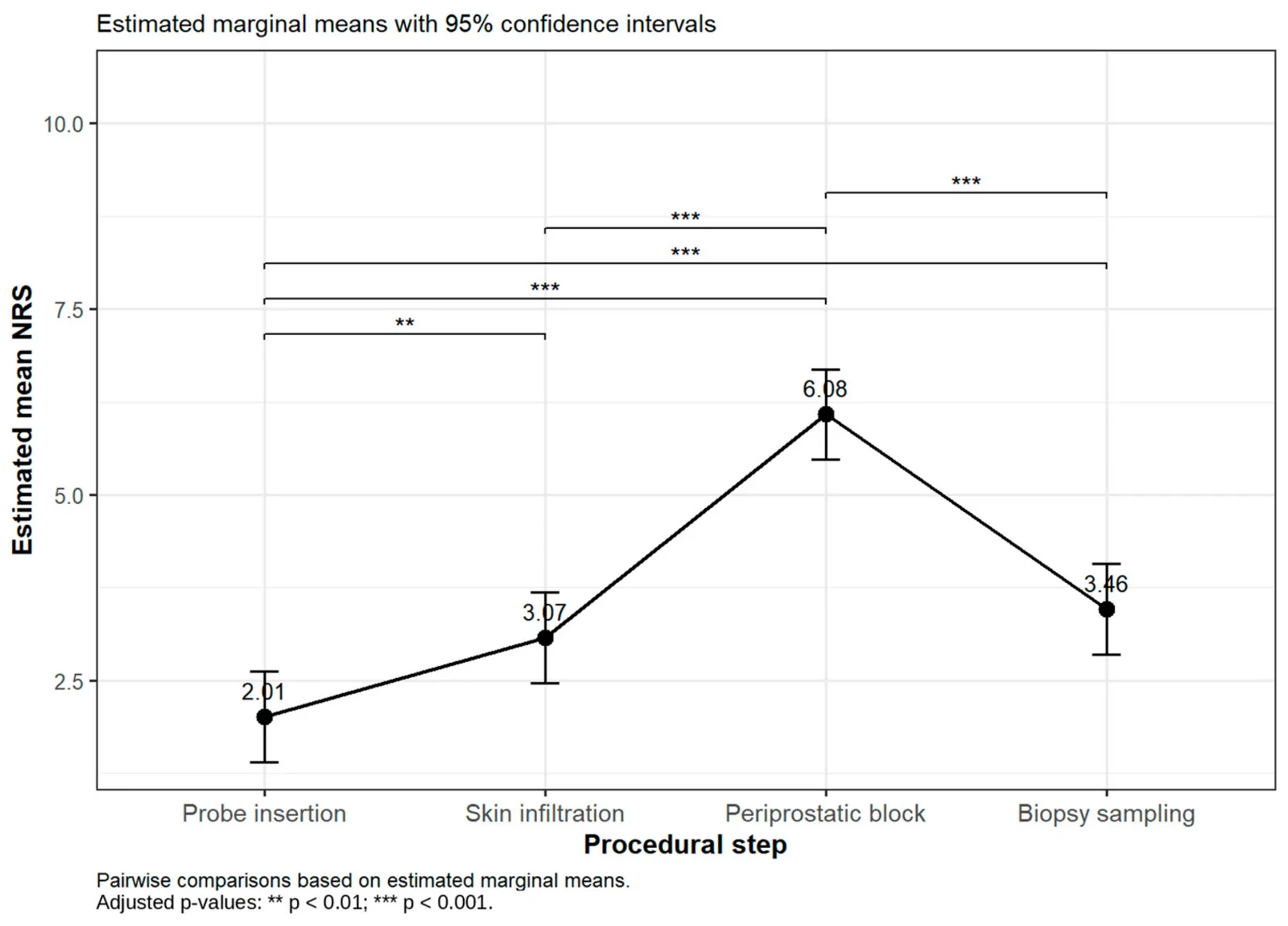
Estimated marginal means with 95% confidence intervals derived from the repeated-measures model; brackets denote pairwise comparisons adjusted using the Holm method (** *p* < 0.01 and *** *p* < 0.001). Pain peaked sharply at administration of the periprostatic nerve block and declined during core acquisition. Repeated-measures ANOVA with Greenhouse–Geisser correction: F(2.68, 472.39) = 230.51, *p* < 0.001, partial η^2^ = 0.567. NRS, Numeric Rating Scale (0–10).

Post hoc comparisons with Holm adjustment confirmed that the periprostatic block was more painful than probe insertion (mean difference of 4.07 points, 95% CI 3.30–4.85), skin infiltration (3.01, 2.23–3.78) and biopsy sampling (2.62, 1.85–3.40; all *p* < 0.001). Probe insertion was the least painful step, scoring lower than skin infiltration (1.06, 0.29–1.84; *p* = 0.009) and biopsy sampling (1.45, 0.67–2.22; *p* < 0.001). Skin infiltration and biopsy sampling did not differ (0.39, −0.39 to 1.16; *p* = 0.329). Estimated marginal means with pairwise comparisons are shown in Figure 6.

Pain therefore rose from probe insertion through skin infiltration to a distinct peak at the periprostatic block and then fell during core acquisition to a level comparable with skin infiltration.

### 3.4. Univariable Predictors of Maximum Procedural Pain

In univariable analysis, three predictors were significantly associated with maximum NRS score: pre-biopsy state anxiety (β = 0.06 per point, 95% CI 0.03–0.08; *p* < 0.001; R^2^ = 0.109), midline perineal tissue thickness (β = 0.26 per cm, 95% CI 0.10–0.43; *p* = 0.002; R^2^ = 0.054) and prostate volume (β = 0.02 per mL, 95% CI 0.01–0.03; *p* = 0.003; R^2^ = 0.050). All three remained significant after Benjamini–Hochberg adjustment (q ≤ 0.012). Body mass index showed a positive association that did not reach significance (β = 0.07 per unit, 95% CI −0.01 to 0.15; *p* = 0.071). Age, PSA, number of cores, number of prior biopsies, prior biopsy status, anterior lesion location and levator ani thickness were not associated with pain (all *p* > 0.35). Each predictor accounted for a small proportion of variance individually, with anxiety explaining the largest share (Table 3).

### 3.5. Multivariable Regression

**Figure 7:**
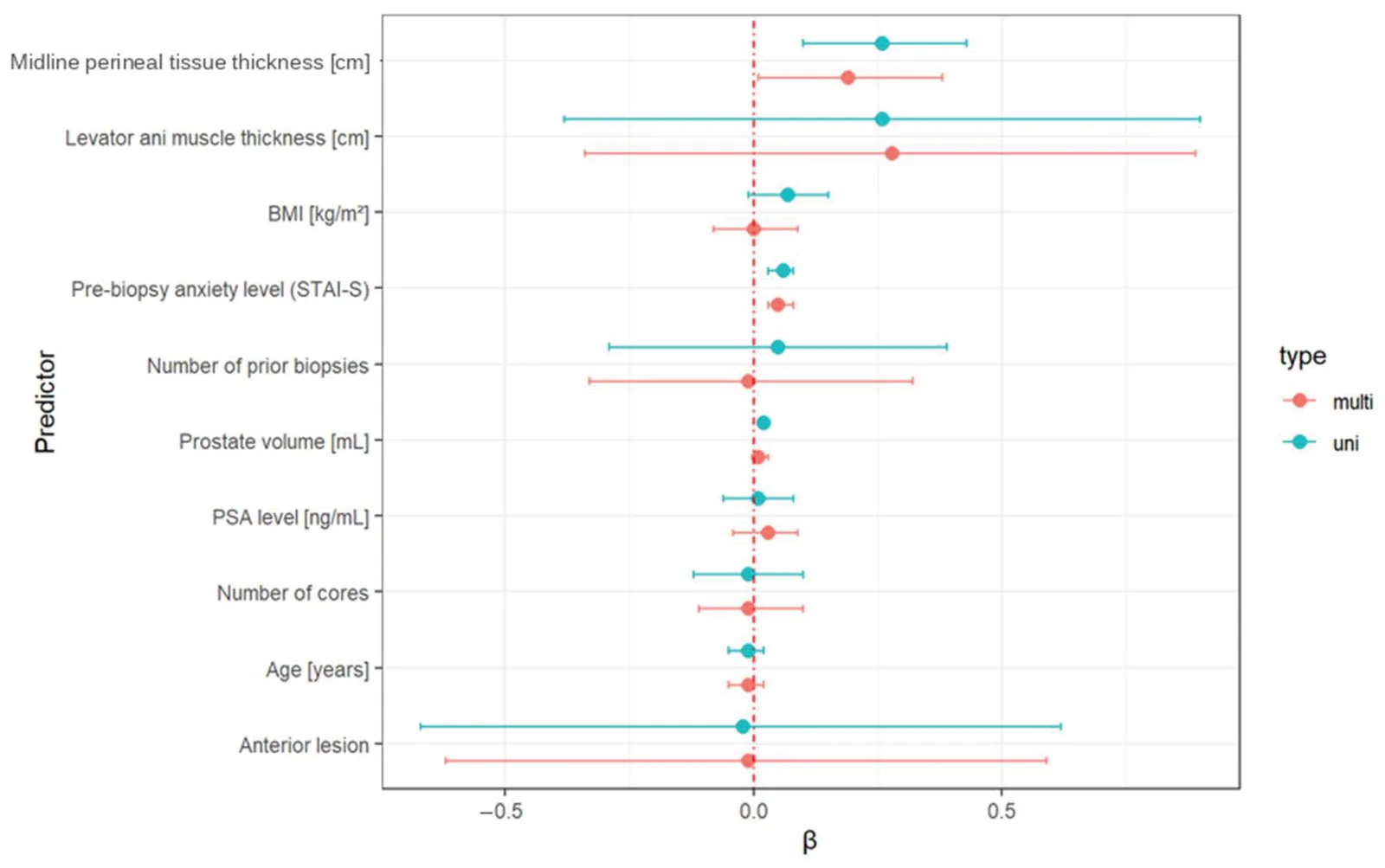
Univariable and multivariable associations of candidate predictors with maximum NRS score during transperineal MRI-fusion prostate biopsy (n = 177).

All ten candidate predictors were entered simultaneously into a linear regression model for maximum NRS score, without stepwise selection. Results are shown alongside the corresponding univariable estimates in Table 3 and Figure 7.

The model explained 19.2% of the variance in maximum pain (adjusted R^2^ = 0.144). Pre-biopsy state anxiety was the strongest independent predictor (B = 0.05 per point, 95% CI 0.03–0.08; standardised β = 0.30; partial R^2^ = 0.094; *p* < 0.001), followed by prostate volume (B = 0.02 per mL, 95% CI 0.00–0.03; β = 0.19; partial R^2^ = 0.036; *p* = 0.014). Midline perineal tissue thickness showed only a borderline association after adjustment, with a 95% CI including the null (B = 0.19 per cm, 95% CI −0.00 to 0.38; β = 0.17; partial R^2^ = 0.023; *p* = 0.052). Levator ani thickness, body mass index, age, PSA, number of cores, number of prior biopsies and anterior lesion location were not associated with pain, each accounting for less than 1% of unique variance. All variance inflation factors were below 1.5, indicating no problematic collinearity.

The attenuation of the body mass index effect on adjustment is consistent with its moderate correlation with midline perineal tissue thickness, which reflects an overlapping aspect of body habitus.

Anxiety and prostate volume thus retained their direction, magnitude and statistical significance after adjustment, indicating that their associations with pain were largely independent of the remaining covariates. The association of midline perineal tissue thickness was attenuated and is regarded as exploratory. These are conditional associations within the fitted model and do not establish causation. Conversely, the absence of significance for the remaining predictors does not exclude an association, since it may reflect imprecision of estimation, a restricted range of values, collinearity, or a non-linear relationship not captured by the model.

### 3.6. Willingness to Repeat the Procedure

Of 177 patients, 155 (87.6%; 95% Wilson CI 81.9–91.6%) indicated they would undergo the same procedure again if clinically indicated. Willingness to repeat was inversely associated with pain intensity: the odds of accepting a repeat procedure fell with each additional point of maximum NRS score (OR 0.67 per point, 95% CI 0.51–0.88; *p* = 0.003), and the mean maximum NRS score was 5.8 among those willing versus 7.2 among those unwilling (Mann–Whitney *p* = 0.002). Willingness to repeat was therefore high but not independent of the pain experienced. Because 88.7% of patients reported clinically significant pain (NRS score ≥ 4), willingness to repeat should not be interpreted as a measure of analgesic adequacy; it may also reflect the perceived diagnostic importance of the biopsy, the lack of alternatives, communication with the clinician, or relief that the procedure had been completed.

### 3.7. Inter-Rater Reliability of Anatomical Measurements

Inter-rater reliability was assessed on a random subset of 18 cases measured independently by a second radiologist blinded to the first set of measurements. Agreement was excellent for midline perineal tissue thickness (ICC(2,1) 0.93, 95% CI 0.81–0.97) and for levator ani thickness calculated as the mean of both sides (0.90, 95% CI 0.69–0.97), the two parameters entered into the regression models. Agreement for the individual sides was good: 0.81 (95% CI 0.54–0.93) on the right and 0.90 (95% CI 0.74–0.96) on the left. Bland–Altman analysis showed a small systematic difference, with the second rater recording slightly higher values than the first (mean difference of −0.25 cm, 95% limits of agreement of −1.48 to +0.97 for midline perineal tissue thickness; −0.14 cm, −0.57 to +0.30 for levator ani thickness).

## 4. Discussion

### 4.1. Principal Findings

In this prospective, pre-registered cohort of 177 men undergoing transperineal MRI-fusion prostate biopsy under local anaesthesia, procedural pain was common and frequently substantial. Clinically significant pain (maximum NRS score ≥ 4) was reported by 88.7% of patients, and the periprostatic nerve block emerged unambiguously as the painful peak (mean NRS score of 5.88 ± 2.03), exceeding every other procedural step, including acquisition of the biopsy cores.

Two variables were independently associated with maximum pain: pre-procedural state anxiety (standardised β = 0.30; *p* < 0.001) and prostate volume (β = 0.19; *p* = 0.014). Midline perineal tissue thickness, significant in univariable analysis, was attenuated after adjustment (*p* = 0.052) and is regarded as exploratory, while levator ani thickness was unrelated to pain in either analysis. Neither the number of biopsy cores nor body mass index predicted pain once other factors were accounted for.

### 4.2. Pre-Procedural Anxiety as the Leading Predictor

Pre-procedural state anxiety was the strongest independent predictor of procedural pain, accounting for the largest unique share of explained variance (partial R^2^ = 0.094), approximately 2.6-fold that of prostate volume and close to half of the variance explained by the full model.

This is consistent with the literature linking anticipatory psychological state to heightened pain perception. State anxiety, together with pain catastrophising, expected pain intensity and negative affect, was identified as a probable predictor of postoperative pain intensity [10], and pre-operative anxiety is among the most consistently reproduced predictors of both pain intensity and analgesic consumption [11].

Evidence specific to prostate biopsy is sparser. Nakai et al. found pre-procedural anxiety to be associated with greater pain during transrectal biopsy performed without anaesthesia [15].

Mechanistically, emotional state and attentional focus modulate nociceptive processing through partially distinct cortical and brainstem descending pathways, such that negative affect and anticipatory arousal lower the threshold at which afferent input is experienced as painful [16,17]. An additional peripheral contribution through anticipatory pelvic floor muscle tension is biologically plausible in the transperineal setting, although muscle activity was not assessed and levator ani thickness was unrelated to pain.

Clinically, the strongest predictor identified here is also the only modifiable one. Prostate volume and perineal anatomy are fixed patient characteristics, whereas anxiety is amenable to structured pre-procedural counselling, explicit procedural information and, in selected patients, pharmacological anxiolysis. Whether anxiety-directed strategies translate into measurably lower procedural pain remains to be established prospectively.

State anxiety fell substantially between the pre-procedural assessment and the measurement obtained after biopsy (43.3 ± 10.9 to 36.4 ± 10.6 STAI-S points; mean difference 6.9 points; *p* < 0.001), with a moderate-to-large effect size (Hedges’ g = 0.70; 95% CI 0.54–0.87). The direction of change was predominant but not uniform: anxiety increased in 15.3% of patients, and the post-procedural distribution retained a longer upper tail than the pre-procedural one (maximum of 79 versus 68 points).

The same variable that fell after the procedure was the strongest predictor of the pain experienced during the procedure. Together, these results identify the pre-procedural interval as the period in which anxiety is the highest and therefore the most accessible to intervention.

This interpretation must remain provisional. Without a control condition, the observed decline cannot be attributed to the procedure itself, since regression to the mean and the natural resolution of anticipatory arousal are equally compatible with the data.

### 4.3. Prostate Volume

Prostate volume was the second independent predictor of maximum pain (B = 0.02 per mL; standardised β = 0.19; *p* = 0.014), corresponding to approximately 0.2 NRS points per additional 10 mL of gland volume.

Gland size may compromise the efficacy of the periprostatic block, which has been shown to be attenuated by larger prostate volume [18]. This is of particular relevance here, given that the block was the most painful step in our cohort.

A sampling-burden explanation is not supported by our data. Although core number has been reported to be independently associated with pain during transperineal biopsy under local anaesthesia [14], the number of cores showed no association with maximum pain in either univariable or multivariable analysis (B = −0.01; *p* = 0.868). The volume–pain association therefore appears to operate through mechanisms other than the extent of sampling.

Evidence on gland size and biopsy pain has been inconsistent. Sonmez et al. identified larger prostate volume among the parameters correlating with VAS scores in transrectal fusion biopsy, with a reported threshold of 61.6 mL [12]. Our data support a modest but independent contribution across the full range of gland sizes, without evidence of a discrete threshold effect.

The practical value of this finding lies in its availability: prostate volume is routinely known before biopsy from MRI, requires no additional assessment, and can be combined with a brief anxiety measure into straightforward pre-procedural risk stratification.

### 4.4. MRI-Derived Perineal Anatomical Parameters

Midline perineal tissue thickness was associated with maximum pain in univariable analysis (B = 0.26 per cm; *p* = 0.002), but the adjusted estimate was borderline, with a 95% CI including the null (B = 0.19 per cm; β = 0.17; *p* = 0.052). We therefore regard it as an exploratory association involving a midline anatomical surrogate, pending confirmation with route-specific measurements.

The attenuation on adjustment is consistent with the moderate correlation between midline perineal tissue thickness and body mass index, which describe overlapping aspects of body habitus. The transrectal literature has approached anatomy through parameters describing the rectal access corridor, such as prostate–anus distance and anorectal angle [12], which have no counterpart in the transperineal route.

An important limitation of this measurement is that it was obtained in the midline sagittal plane, whereas both the periprostatic block and the targeted and perilesional cores follow paramedian, bilateral and target-dependent trajectories. Midline perineal tissue thickness is therefore a surrogate of perineal soft-tissue bulk rather than a measurement of the path actually traversed by the needle. Its excellent inter-rater reliability shows that it can be measured consistently, but reproducibility does not establish anatomical validity for the needle path. Template coordinates of individual needle passes were not recorded, which precluded a route-specific analysis; bilateral paramedian skin-to-capsule distances at the block entry sites would be a more anatomically congruent exposure for future studies.

Levator ani thickness was unrelated to pain in either analysis (B = 0.24 per cm; *p* = 0.454), despite good reproducibility (ICC 0.90). A contribution of the pelvic floor remains biologically plausible; however, static muscle thickness is not the relevant quantity: tone rather than bulk may be operative, and anticipatory pelvic floor tension is not captured by an anatomical measurement obtained before the procedure. Dynamic assessment, by electromyography at the time of biopsy, would be needed to test this.

### 4.5. The Periprostatic Block as the Painful Peak of the Procedure

The most consistent finding of this study was the dominance of the periprostatic nerve block as the source of procedural pain. Mean pain during the block (NRS score of 5.88 ± 2.03) exceeded that of every other step by a wide margin, and procedural step accounted for the majority of within-patient variance in reported pain (partial η^2^ = 0.567). The block was more painful than core acquisition (mean difference of 2.62 points; 95% CI 1.85–3.40; *p* < 0.001), a difference exceeding the commonly accepted threshold for a clinically meaningful change in the NRS score, and it was the only step at which no patients reported a score of zero. The same pattern has been described in the largest transperineal fusion series reported to date, in which pain during administration of local anaesthesia exceeded pain during core sampling [13].

Efforts to improve tolerability should consequently target this specific moment. The warming and bicarbonate buffering of the local anaesthetic both reduce injection pain in meta-analyses of cutaneous infiltration [19,20], although these estimates derive from superficial injection and their transfer to a deep pelvic block has not been formally tested. A more fundamental alternative is to change the block itself: in a multicentre randomised trial, a perineal nerve block achieved better pain control than a periprostatic block in men undergoing transperineal biopsy [21]. Our pain scores are higher than those reported in the largest series of transperineal biopsy under local anaesthesia. In 1287 consecutive men, Stefanova et al. found the procedure to be well tolerated; among 511 patients with step-wise pain ratings, mean VAS scores were 3.1 for skin infiltration, 3.0 for periprostatic infiltration, 2.5 for core sampling and 2.4 for probe insertion [22]. The pattern matches ours: administration of local anaesthesia, not core sampling, was the most uncomfortable part of the procedure. The difference in absolute pain is likely to reflect technique and assessment. Stefanova et al. used a freehand approach with two perineal access cannulas, which limits the number of skin and pelvic floor punctures, and a larger anaesthetic volume (25 mL of 1% lidocaine); tolerability data were retrieved retrospectively from patient records. In our cohort, template-guided sampling required a separate percutaneous pass for each core, 20 mL of 1% lignocaine was used, and pain was recorded prospectively by an independent nurse. Access technique may therefore matter for tolerability as much as the anaesthetic itself.

### 4.6. Strengths and Limitations

This study has several strengths. The design was prospective with a publicly pre-registered protocol and analysis plan, and all deviations from that plan are documented in a protocol amendment on the Open Science Framework. Enrolment was consecutive, and the Full Analysis Set of 177 men had no missing data on the primary outcome or on any analysed predictor. Anxiety was quantified with a validated instrument rather than a single-item proxy. Pain was recorded separately at four procedural stages, which allowed the peak to be localised rather than assumed, and the reproducibility of the MRI measurements was formally assessed with a second independent reader.

Several limitations temper the conclusions. The model explained a modest proportion of variance (R^2^ = 0.192; adjusted R^2^ = 0.144), indicating that most between-patient variation in pain remains unexplained. Neither pain catastrophising nor expected pain was measured; because both are established correlates of procedural pain and of anxiety, the residual confounding of the anxiety effect cannot be excluded, and our data cannot establish whether patients over-anticipated the discomfort that they subsequently reported. Midline perineal tissue thickness reached only borderline significance and is a midline surrogate rather than a route-specific measurement. The pre-specified logistic analysis of clinically significant pain could not be performed, since the observed event rate of 88.7% left too few patients in the reference group. Our sampling protocol comprised targeted and perilesional cores only (median of 9, IQR 7–12), without systematic sampling. In daily practice, targeted cores are often combined with systematic and perilesional cores, and extended schemes may reach 18 cores or more [23]. Core number was not associated with pain within the range observed here, but this cannot be extrapolated beyond it; pain during combined or extended sampling under local anaesthesia may therefore be higher than reported in this study. This study was also conducted at a single centre with one anaesthetic protocol and three operators, which may limit generalisability. Because the operator was not recorded for individual procedures, operator-related variation in block delivery could not be examined and may have contributed to between-patient differences in pain. Finally, the design was observational: the associations reported here are conditional within the fitted model and do not establish causation, and the proposed pathway from body habitus through perineal tissue thickness to pain remains a hypothesis to be tested prospectively.

### 4.7. Clinical Implications

Two variables available before biopsy: state anxiety and prostate volume were independently associated with procedural pain. Both are obtainable without additional burden: prostate volume is already reported on the diagnostic MRI, and state anxiety can be captured with a brief validated instrument while the patient waits. This supports pre-procedural risk stratification, in which men identified as being at higher risk are offered enhanced measures such as structured counselling, an optimised or alternative block technique, or in selected cases, anxiolysis.

The finding with the most immediate practical consequence, however, concerns the anaesthetic itself. Clinically significant pain was reported by 88.7% of patients, and the periprostatic block was the single most painful step in the procedure.

Local anaesthesia is not the only option. In high-volume centres, transperineal biopsy is often performed under sedation, particularly when extensive sampling is planned, as in saturation biopsy combined with fusion-targeted cores in men on active surveillance or in those requiring repeat biopsy; sedation reduces procedural pain and has been advocated to improve the quality of the cores obtained [24]. Our data do not compare the two approaches, but they help identify men in whom sedation should be considered: those with high pre-procedural anxiety, those with large glands, and those in whom the planned number of cores substantially exceeds the targeted and perilesional sampling studied here.

Two caveats bound how far these findings can be taken. The model explained approximately one-fifth of the variance in maximum pain, which supports inference about group-level determinants but not individual-level prediction; discrimination and calibration were not assessed, and the model has not been externally validated, so any stratification algorithm derived from these data would require prospective evaluation before clinical use. Of the two predictors, moreover, only anxiety is modifiable, and whether reducing it lowers procedural pain is a causal claim that this design cannot support. These findings are therefore best read as identifying candidate targets for intervention rather than as providing a ready-to-use clinical tool.

## 5. Conclusions

In men undergoing transperineal MRI-fusion prostate biopsy under local anaesthesia, procedural pain was common and peaked sharply at administration of the periprostatic nerve block rather than at acquisition of the biopsy cores. Clinically significant pain was reported by 88.7% of patients, indicating that current local anaesthetic protocols leave most men with meaningful discomfort.

Pre-procedural state anxiety was the strongest independent predictor of maximum pain, followed by prostate volume. Midline perineal tissue thickness showed only an exploratory association of borderline significance, while levator ani thickness was unrelated to pain. Neither the number of biopsy cores nor body mass index predicted pain after adjustment.

Pre-procedural anxiety should be assessed and addressed with structured information and counselling, and with pharmacological anxiolysis in selected men. Furthermore, because the periprostatic block is the painful peak, the block itself should be optimised. Sedation should be considered in men with high anxiety, a large gland, or a planned extensive sampling scheme, such as saturation or repeat biopsy in active surveillance. Anterior lesion location was not associated with pain in our cohort. The choice of anaesthesia should rest on anxiety, gland volume and the planned extent of sampling rather than on lesion location alone. Willingness to undergo the procedure again was high, but was inversely related to the pain experienced and should not be equated with comfort.

## Figures and Tables

**Table 1:** Baseline demographic, clinical, and procedural characteristics of the study cohort (*n* = 177).

Characteristic	Value (n = 177)
**Demographic characteristics**	
Age, years	64.6 ± 8.6
Body mass index, kg/m^2^	27.0 [25.0–29.0]
**Clinical characteristics**	
PSA, ng/mL	5.8 [4.5–8.3]
Prostate volume, mL	48 [34–66]
Biopsy history, n (%)	
Biopsy-naïve	122 (68.9)
Previous biopsy	55 (31.1)
Pre-procedural anxiety, STAI-S score	43.3 ± 10.9
**MRI-derived anatomical measurements**	
Midline perineal tissue thickness (MPT), cm	7.9 [6.9–9.0]
Levator ani thickness, right, cm	0.93 [0.67–1.25]
Levator ani thickness, left, cm	0.95 [0.72–1.25]
Levator ani thickness (LAT), mean of both sides, cm	0.95 [0.71–1.24]
**Procedural characteristics**	
Cores obtained, n	9 [7–12]

Bold rows are category headings and contain no data.

**Table 2:** Pain intensity at each procedural step of transperineal MRI-fusion prostate biopsy (n = 177).

Characteristic	Overall N = 708	Probe Insertion N = 177	Skin Infiltration N = 177	Periprostatic Block N = 177	Biopsy Sampling N = 177
NRS score					
Min	0.00	0.00	0.00	1.00	0.00
Mean (±SD)	3.66 (±2.30)	2.21 (±1.91)	3.22 (±1.57)	5.88 (±2.03)	3.31 (±1.93)
Max	10.00	8.00	8.00	10.00	9.00

**Table 3:** Univariable and multivariable linear regression analysis.

		Univariable		Multivariable			Adjusted Predictor Importance		
Predictor	Comparison	B (95% CI)	*p*	B (95% CI)	β Stand.	*p*	Partial R^2^	VIF/Adj. GVIF	N (UV)
Number of cores	Per 1-unit increase	−0.01 (−0.12; 0.10)	0.828	−0.01 (−0.12; 0.10)	−0.01	0.868	0.000	1.07	177
PSA level [ng/mL]	Per 1-unit increase	0.01 (−0.06; 0.08)	0.712	0.02 (−0.05; 0.09)	0.04	0.633	0.001	1.19	177
Age [years]	Per 1-unit increase	−0.01 (−0.05; 0.02)	0.402	−0.02 (−0.05; 0.01)	−0.08	0.291	0.007	1.07	177
BMI [kg/m^2^]	Per 1-unit increase	0.07 (−0.01; 0.15)	0.071	0.02 (−0.07; 0.10)	0.03	0.724	0.001	1.38	177
Number of prior biopsies	Per 1-unit increase	0.05 (−0.29; 0.39)	0.763	−0.01 (−0.34; 0.32)	−0.00	0.954	0.000	1.13	177
Prostate volume [mL]	Per 1-unit increase	0.02 (0.01; 0.03)	0.003	0.02 (0.00; 0.03)	0.19	0.014	0.036	1.20	177
Pre-biopsy anxiety level (STAI-S)	Per 1-unit increase	0.06 (0.03; 0.08)	<0.001	0.05 (0.03; 0.08)	0.30	<0.001	0.094	1.05	177
Midline perineal tissue thickness [cm]	Per 1-unit increase	0.26 (0.10; 0.43)	0.002	0.19 (−0.00; 0.38)	0.17	0.052	0.023	1.48	177
Levator ani muscle thickness [cm]	Per 1-unit increase	0.26 (−0.38; 0.90)	0.423	0.24 (−0.39; 0.86)	0.06	0.454	0.003	1.11	177
Anterior lesion							0.000	1.04	177
	1 (reference)								
	0 vs. 1	−0.02 (−0.67; 0.62)	0.941	−0.02 (−0.64; 0.59)	—	0.937			

Multivariable model: N = 177; R^2^ = 0.192; adjusted R^2^ = 0.144. UV = univariable model; MV = multivariable model; B = unstandardised regression coefficient; β stand. = standardised regression coefficient for continuous predictors in the multivariable model; Partial R^2^ = proportion of variance uniquely associated with the predictor after adjustment for all other predictors; VIF is shown for 1-df predictors and adjusted GVIF = GVIF^(1/(2 × df)) for predictors with >1 df. Reference categories are indicated explicitly.

## Data Availability

The pre-registered protocol is available at https://osf.io/pbacu (accessed on 27 September 2026). The anonymised dataset is available at https://osf.io/cfejh (accessed on 27 September 2026).
